# Supplementary material for: Identification of a new gibberellin receptor agonist, diphegaractin, by a cell-free chemical screening system
Source: Commun Biol. 2023 May 9;6:448. doi: 10.1038/s42003-023-04760-y (PMC10170162; doi:10.1038/s42003-023-04760-y)
Supplement: Supplementary file 2 — Supplementary Information [file 42003_2023_4760_MOESM2_ESM.pdf]

Identification of a new gibberellin receptor agonist, diphegaractin, by a cell-free chemical screening system

Akira Nozawa[1], Ryoko Miyazaki[1], Yoshinao Aoki[2], Reina Hirose[1], Ryosuke Hori[1], Chihiro Muramatsu[1], Yukinori Shigematsu[3], Keiichirou Nemoto[4], Yoshinori Hasegawa[5], Keiko Fujita[6], Takuya Miyakawa[7], Masaru Tanokura[7], Shunji Suzuki[2], Tatsuya Sawasaki[1,*]

[1]*Proteo-Science Center, Ehime University, 3 Bunkyo-cho, Matsuyama, Ehime 790-8577, Japan.*

[2]*The Institute of Enology and Viticulture, University of Yamanashi, 1-13-1, Kitashin, Kofu, Yamanashi 400-0005, Japan.*

[3]*Fruit Tree Research Center, Ehime Research Institute of Agriculture, Forestry and Fisheries, 1618 Shimo-idai, Matsuyama, Ehime 791-0112, Japan.*

[4]*Iwate Biotechnology Research Center, 22-174-4 Narita, Kitakami, Iwate 024-0003, Japan.*

[5]*Department of Applied Genomics, Kazusa DNA Research Institute, 2-6-7 Kazusa-kamatari, Kisarazu, Chiba 292-0818, Japan.*

[6]*Faculty of Bioresource Sciences, Prefectural University of Hiroshima, 5562 Nanatsuka-cho, Shobara, Hiroshima 727-0023, Japan.*

[7]*Department of Applied Biological Chemistry, Graduate School of Agricultural and Life Sciences, The University of Tokyo, 1-1-1 Yayoi, Bunkyo-ku, Tokyo 113-8657, Japan.*

***Corresponding author:**

Tatsuya Sawasaki

Proteo-Science Center,

Ehime University, Matsuyama 790-8577, Japan

Tel: 81-89-927-8530

Fax: 81-89-927-9941

E-mail: sawasaki@ehime-u.ac.jp

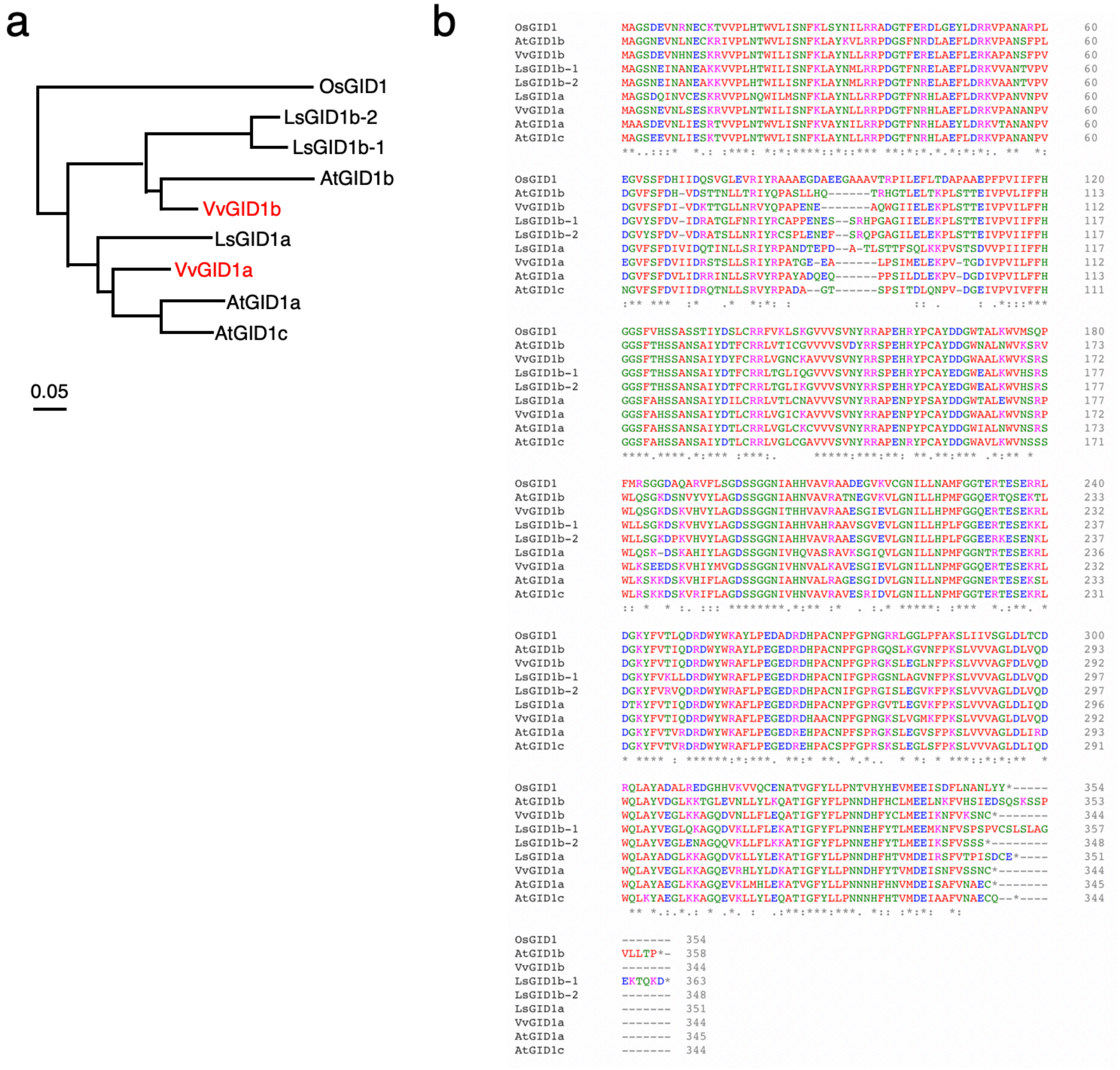

Supplementary Figure 1

Phylogenetic tree and amino acid sequences of GID1 proteins. a, Phylogenetic tree of GID1 proteins from grape, *Arabidopsis*, lettuce, and rice. The dendrogram indicates the relative evolutionary distances among GID1 proteins and was generated using the UPGMA method. Bar indicates the genetic distance for 0.05 amino acid substitutions/site. **b**, Amino acid sequence alignment of GID1 proteins.

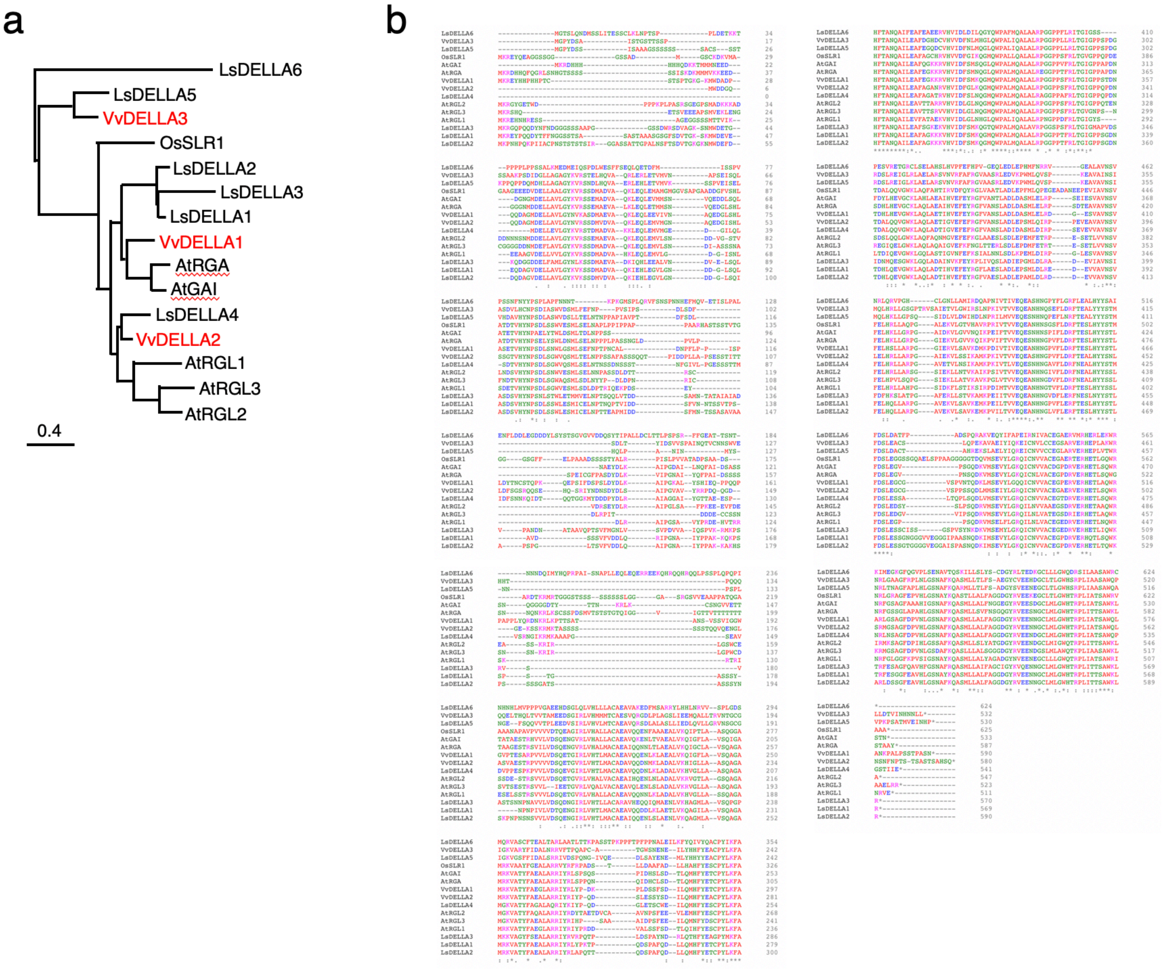

Supplementary Figure 2

Phylogenetic tree and amino acid sequences of the DELLA1 proteins. a, Phylogenetic tree of DELLA proteins from grape, *Arabidopsis*, lettuce, and rice. The dendrogram indicates the relative evolutionary distances among the DELLA proteins and was generated using the UPGMA method. Bar indicates the genetic distance for 0.4 amino acid substitutions/site. **b**, Amino acid sequence alignment of DELLA proteins.

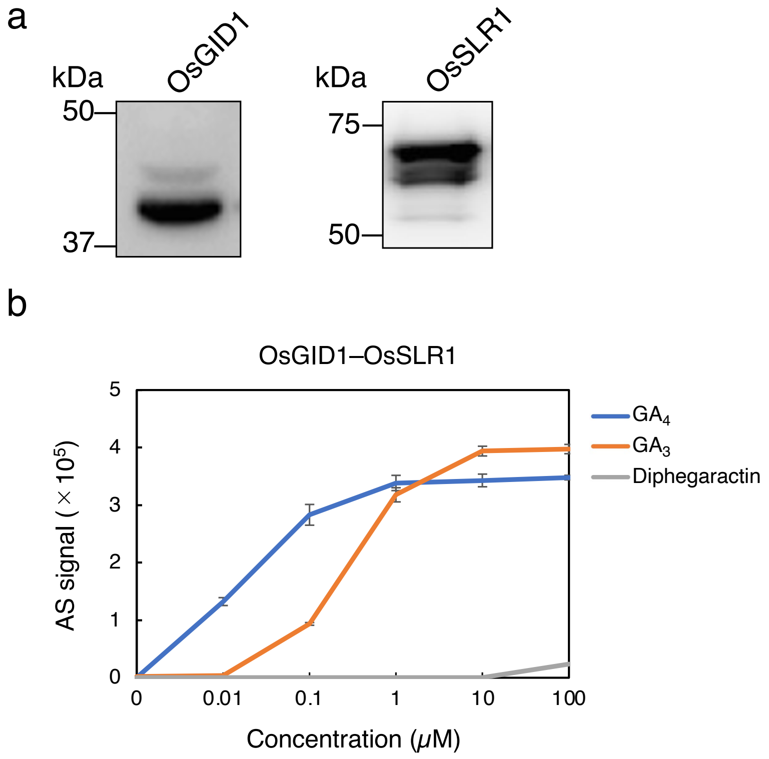

Supplementary Figure 3

***In vitro* interaction analysis of GID1 and DELLA proteins from rice. a**, Synthesis of OsGID1 and OsSLR. Biotinylated OsGID1 and FLAG-tagged OsSLR were synthesised using a wheat cell-free system. The synthesis of these proteins was confirmed by immunoblotting using anti-biotin and anti-FLAG antibodies. **b**, Interaction assay between OsGID1 and OsSLR The interaction between OsGID1 and OsSLR was analysed using the AlphaScreen system using GA$_3$, GA$_4$, and diphegaractin at various concentrations. Data are shown as mean ± standard deviation from three independent experiments.

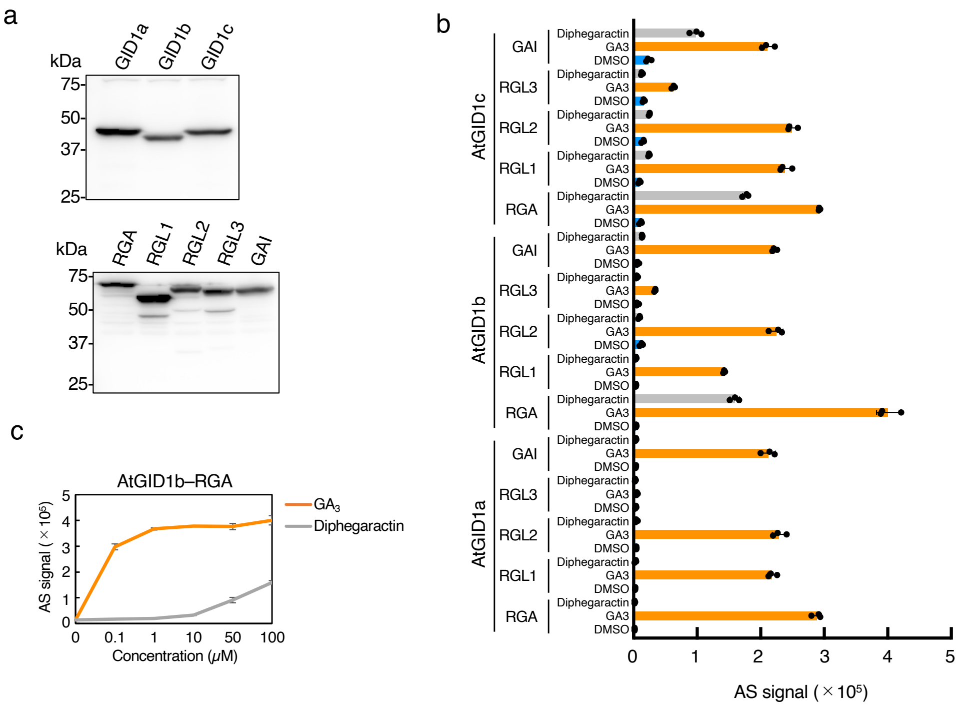

Supplementary Figure 4

***In vitro* interaction analysis of GID1 and DELLA proteins from *Arabidopsis*. a**, Synthesis of AtGID1 and AtDELLA proteins. Biotinylated AtGID1 and FLAG-tagged AtDELLA proteins were synthesised using a wheat cell-free system. The synthesis of these proteins was confirmed by immunoblotting using anti-biotin and anti-FLAG antibodies. **b**, Interaction assay between AtGID1 and AtDELLA proteins. Interaction of AtGID1 and AtDELLA proteins was analysed using the AlphaScreen system using GA_3 and diphegaractin at 0 or 100 μM. Data are shown as mean ± standard deviation from three independent experiments. **c,** Interaction assay between AtGID1b and AtRGA The interaction between AtGID1b and AtRGA was analysed using the AlphaScreen system using GA_3 and diphegaractin at various concentrations. Data are shown as mean ± standard deviation from three independent experiments.

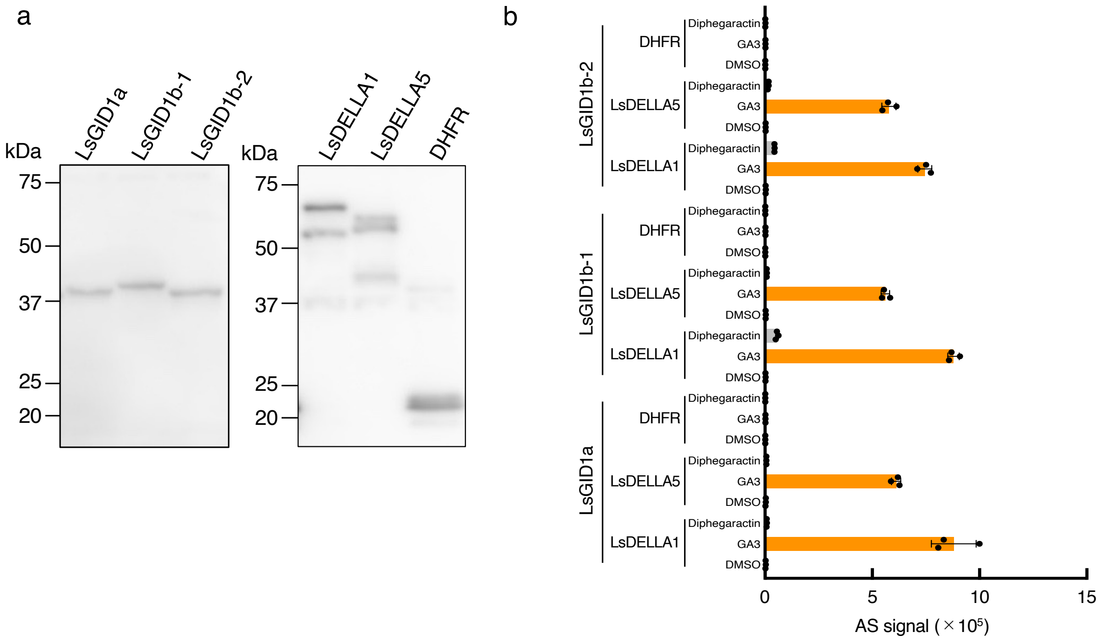

Supplementary Figure 5

***In vitro* interaction analysis of GID1 and DELLA proteins from lettuce. a**, Synthesis of LsGID1 and LsDELLA proteins. Biotinylated LsGID1 and FLAG-tagged LsDELLA proteins were synthesised using a wheat cell-free system. The synthesis of these proteins was confirmed by immunoblotting using anti-biotin and anti-FLAG antibodies. **b**, Interaction assay between LsGID1 and LsDELLA proteins. Interaction of LsGID1 and LsDELLA proteins was analysed using the AlphaScreen system using GA_3 and diphegaractin at 0 or 50 μM. Data are shown as mean ± standard deviation from three independent experiments.

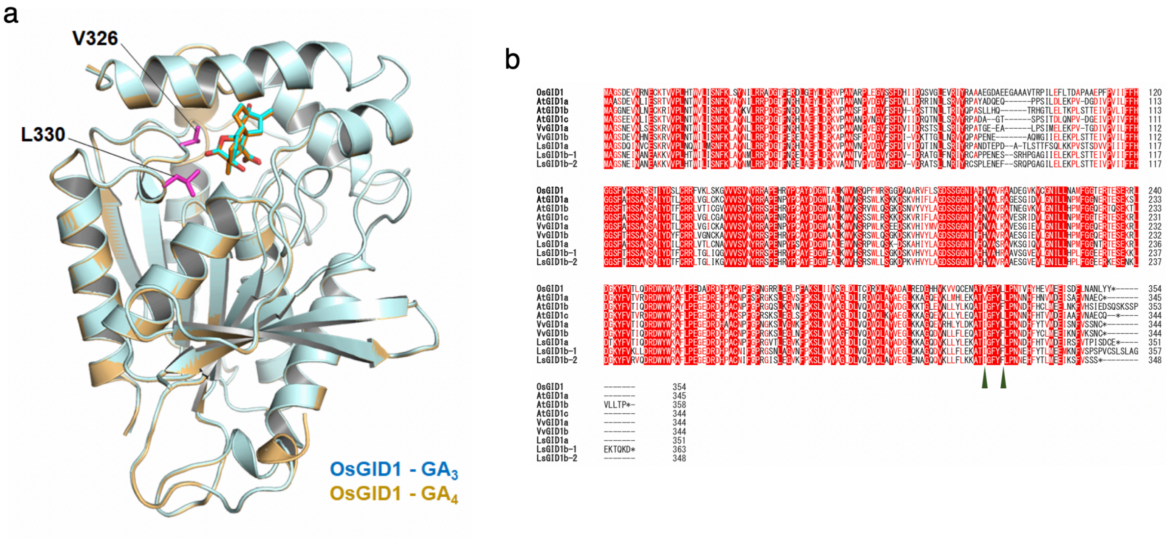

Supplementary Figure 6

Variation in the residues located in the GA-binding pocket. a, Superposed structures of OsGID1 in complex with GA_3 (PDB ID: 3ED1) and GA_4 (PDB ID: 3EBL). The common parts of GA_3 and GA_4 overlapped completely. **b**, Sequence alignment of GID1s. Green triangles indicate the variable residues that correspond to OsGID1 V326 and L330.

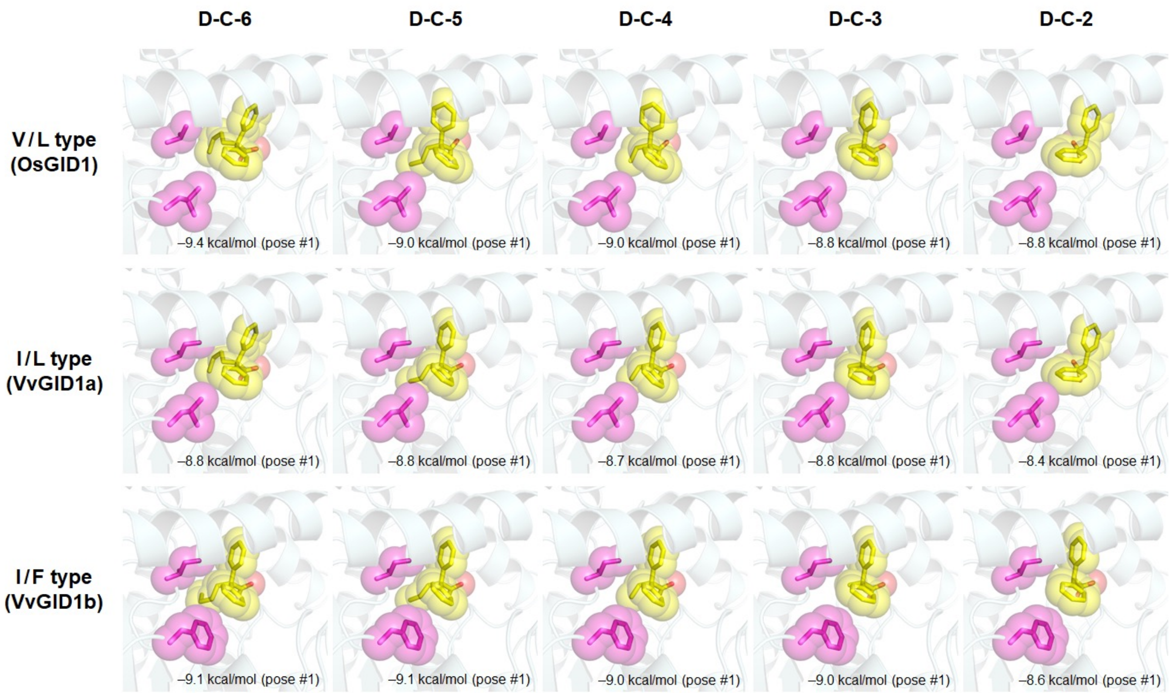

Supplementary Figure 7

Docking models of diphegaractin analogues toward the three GID1 types. Yellow and magenta sticks are diphegaractin analogues and variable residues for the classification of GID1 types, respectively. The affinity score of the best docking pose (#1) is shown at the bottom of each docking model.

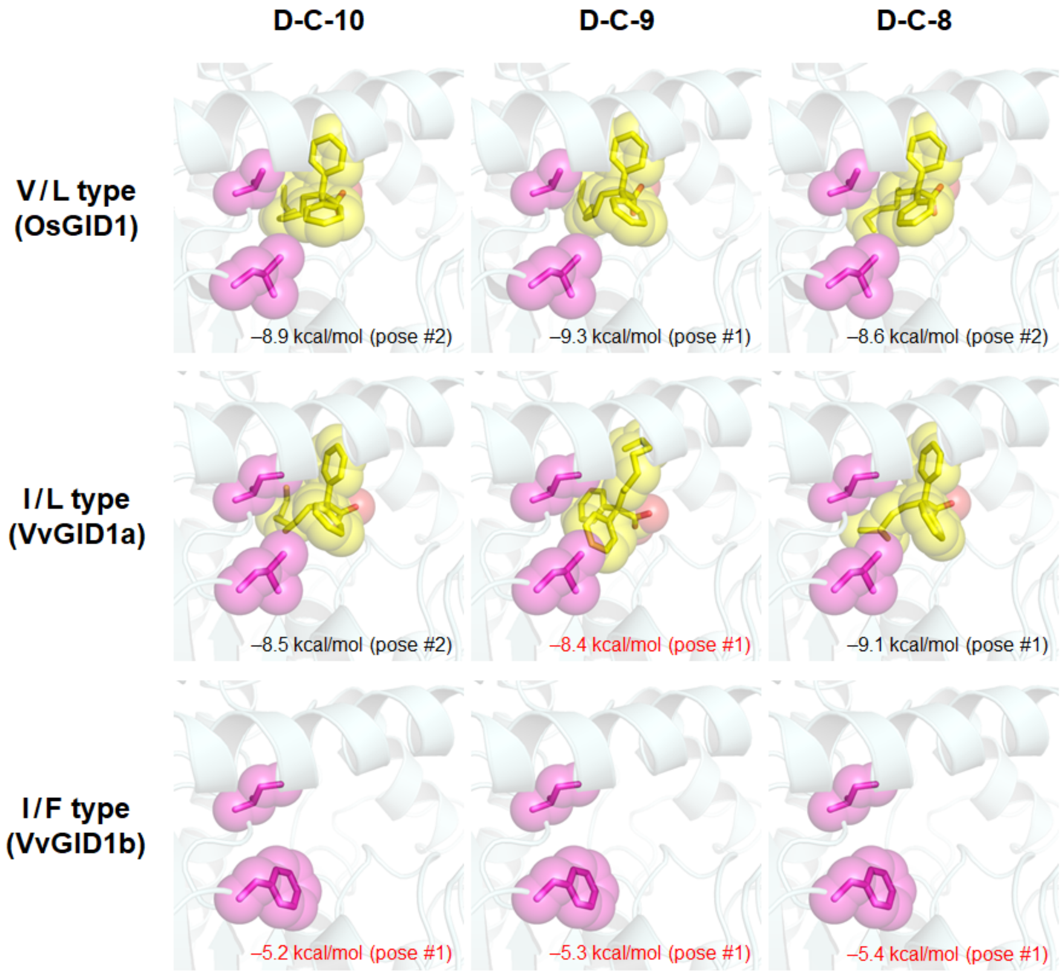

Supplementary Figure 8

Docking models of diphegaractin analogous compounds with a higher alkyl group than diphegaractin toward three GID1 types. Yellow and magenta sticks are diphegaractin analogous compounds and the variable residues for the classification of GID1 types, respectively. Affinity score of the similar docking pose to diphegaraction is shown with black letters at the bottom of each docking model. For models that did not predict docking poses similar to diphegalactin, affinity score of the best docking pose (#1) is shown in red.

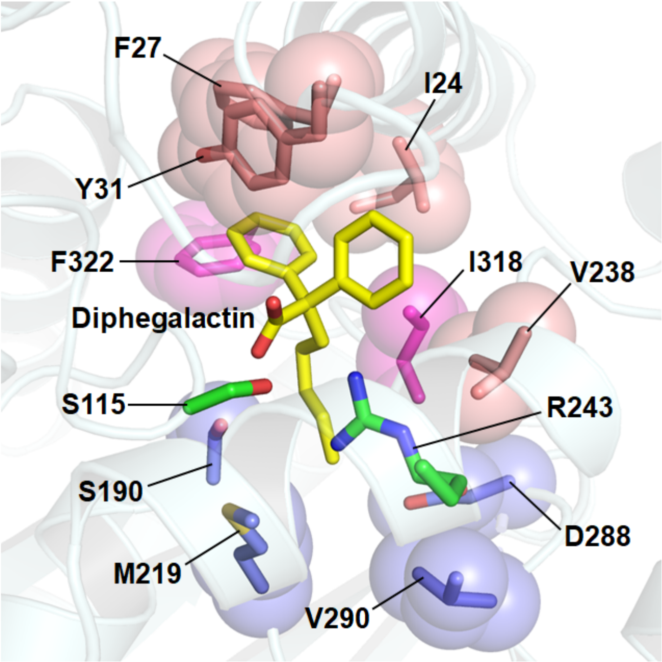

Supplementary Figure 9

Interactions between VvGID1b and diphegalactin. The residues colored in magenta (I/F type) and salmon contribute to hydrophobic interaction with the diphenyl group of diphegalactin (yellow). The alkyl group of diphegalactin is arranged in the space enclosed by the residues colored in magenta and slate. The carboxyl group of diphegalactin is positioned at a distance that allows hydrogen bonds with the side chains of S155 and R243.

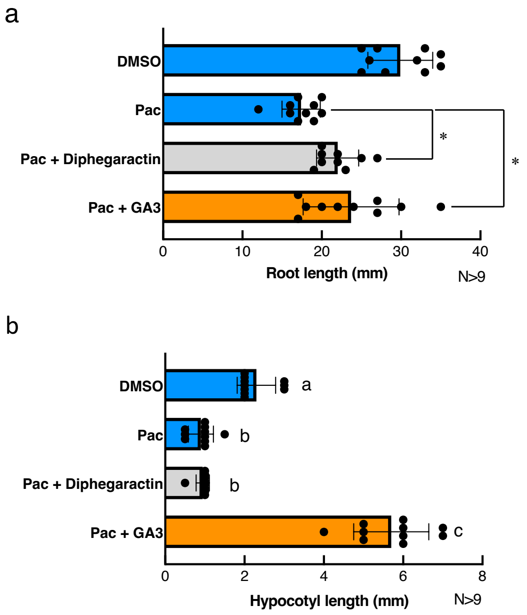

Supplementary Figure 10

Biological analysis of diphegaractin in *Arabidopsis*. a, Effect of diphegaractin on root length of *Arabidopsis*. **b**, Effect of diphegaractin on the hypocotyl length of *Arabidopsis*. The *Arabidopsis* seeds were sown on 1/2MS plate containing 10 μM paclobutrazol (Pac) with or without 50 μM GA_3 or diphegaractin. The plates were incubated vertically at 23°C with a photoperiod of 16 h. After 10 days, the lengths of the root and hypocotyl were measured. Data are shown as mean ± standard deviation from nine or more independent experiments. Asterisks indicate significant differences from the control (* P < 0.05, Student's *t*-test). Different letters indicate statistically significant differences (P < 0.05, Tukey's test).

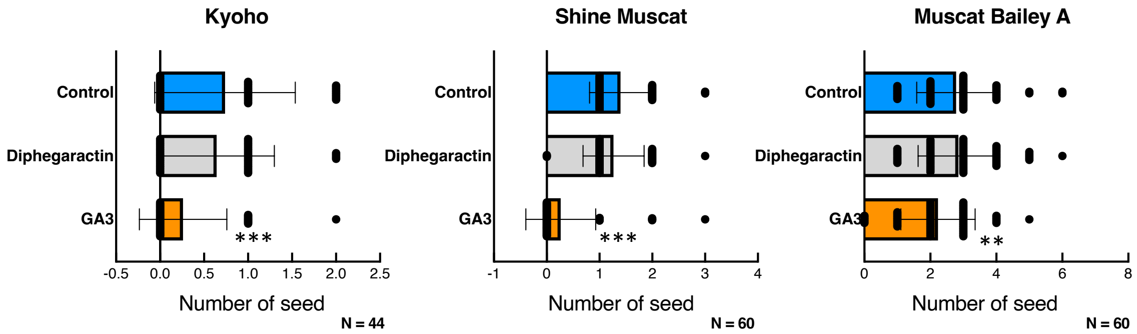

Supplementary Figure 11

Effect of diphegaractin on parthenocarpy of grape berries. Grape spikes were treated with GA_3 or diphegaractin. After 74 d, the number of seeds in each grape berry was measured. Data are shown as mean ± standard deviation from 44 or more independent experiments. Asterisks indicate significant differences from the control (* $P < 0.05$; ** $P < 0.01$; *** $P < 0.001$, Student's *t*-test).
