# Supplementary material for: Identification of a new gibberellin receptor agonist, diphegaractin, by a cell-free chemical screening system
Source: Commun Biol. 2023 May 9;6:448. doi: 10.1038/s42003-023-04760-y (PMC10170162; doi:10.1038/s42003-023-04760-y)
Supplement: Supplementary file 5 — Reporting Summary [file 42003_2023_4760_MOESM5_ESM.pdf]

Corresponding author(s): Tatsuya Sawasaki

Last updated by author(s): Mar 11, 2023

Reporting Summary

Nature Portfolio wishes to improve the reproducibility of the work that we publish. This form provides structure for consistency and transparency in reporting. For further information on Nature Portfolio policies, see our Editorial Policies and the Editorial Policy Checklist.

Statistics

For all statistical analyses, confirm that the following items are present in the figure legend, table legend, main text, or Methods section.

n/a	Confirmed	
☐	☒	The exact sample size (*n*) for each experimental group/condition, given as a discrete number and unit of measurement
☐	☒	A statement on whether measurements were taken from distinct samples or whether the same sample was measured repeatedly
☐	☒	The statistical test(s) used AND whether they are one- or two-sided<br>*Only common tests should be described solely by name; describe more complex techniques in the Methods section.*
☒	☐	A description of all covariates tested
☐	☒	A description of any assumptions or corrections, such as tests of normality and adjustment for multiple comparisons
☐	☒	A full description of the statistical parameters including central tendency (e.g. means) or other basic estimates (e.g. regression coefficient) AND variation (e.g. standard deviation) or associated estimates of uncertainty (e.g. confidence intervals)
☐	☒	For null hypothesis testing, the test statistic (e.g. F, t, r) with confidence intervals, effect sizes, degrees of freedom and P value noted<br>*Give P values as exact values whenever suitable.*
☒	☐	For Bayesian analysis, information on the choice of priors and Markov chain Monte Carlo settings
☒	☐	For hierarchical and complex designs, identification of the appropriate level for tests and full reporting of outcomes
☒	☐	Estimates of effect sizes (e.g. Cohen's *d*, Pearson's *r*), indicating how they were calculated

Our web collection on statistics for biologists contains articles on many of the points above.

Software and code

Policy information about availability of computer code

Data collection	All data collection in this study were performed using softwares attached to each detector.<br>Image Quant LAS 4000 software (GE Healthcare, version 1.1) for chemical luminescent immunoblot.<br>Wallac Envision Manager software (ParkinElmer, version 1.12) for AlphaScreen.
Data analysis	Image analysis was performed using ImageJ (Fiji) software (version 2.1.0).<br>Data analysis and significant changes were performed using Excel (version 16.66) or GraphPad Prism9 (Version 9.5.1).

For manuscripts utilizing custom algorithms or software that are central to the research but not yet described in published literature, software must be made available to editors and reviewers. We strongly encourage code deposition in a community repository (e.g. GitHub). See the Nature Portfolio guidelines for submitting code & software for further information.

Data

Policy information about availability of data

All manuscripts must include a data availability statement. This statement should provide the following information, where applicable:

- Accession codes, unique identifiers, or web links for publicly available datasets
- A description of any restrictions on data availability
- For clinical datasets or third party data, please ensure that the statement adheres to our policy

Source data behind the graphs are available as Supplementary Data 1. Uncropped and unedited blot images are available as Supplementary Fig. 12. RNA sequencing data were deposited to DNA Data Bank of Japan (Accession number: DRA015789, DRA015790, DRA015791).

Human research participants

Policy information about studies involving human research participants and Sex and Gender in Research.

Reporting on sex and gender	N/A
Population characteristics	N/A
Recruitment	N/A
Ethics oversight	N/A

Note that full information on the approval of the study protocol must also be provided in the manuscript.

Field-specific reporting

Please select the one below that is the best fit for your research. If you are not sure, read the appropriate sections before making your selection.

☒ Life sciences ☐ Behavioural & social sciences ☐ Ecological, evolutionary & environmental sciences

For a reference copy of the document with all sections, see nature.com/documents/nr-reporting-summary-flat.pdf

Life sciences study design

All studies must disclose on these points even when the disclosure is negative.

Sample size	For immunoblot analyses, we have chosen more than two independent experiments as sample sizes and traditional experimental approach in biochemical and plant experiments. For quantitative experiments, such as analysis by AlphaScreen , samples were prepared in at least triplicates.
Data exclusions	No data were excluded from analysis.
Replication	All experiments were performed in two or more replicates.
Randomization	No randomization was used in this study as it is not necessary in this experiments.
Blinding	Blinding was not conducted in this study as.

Reporting for specific materials, systems and methods

We require information from authors about some types of materials, experimental systems and methods used in many studies. Here, indicate whether each material, system or method listed is relevant to your study. If you are not sure if a list item applies to your research, read the appropriate section before selecting a response.

Materials & experimental systems

n/a	Involved in the study
☐	☒ Antibodies
☒	☐ Eukaryotic cell lines
☒	☐ Palaeontology and archaeology
☒	☐ Animals and other organisms
☒	☐ Clinical data
☒	☐ Dual use research of concern

Methods

n/a	Involved in the study
☒	☐ ChIP-seq
☒	☐ Flow cytometry
☒	☐ MRI-based neuroimaging

Antibodies

Antibodies used

Anti-FLAG mouse mAb (HRP-conjugated, Sigma-Aldrich, #A8592, 1:5000) and anti-AGIA rabbit mAb (HRP-conjugated, produced in our laboratory, 1:10000) were used to detect epitope-tagged proteins. Biotinylated proteins were detected by anti-biotin goat pAb (HRP-conjugated, Cell Signaling Technology, #7075, 1:3000).Anti-rabbit IgG (HRP-conjugated, Cell Signaling Technology, #7074, 1:10000), anti-mouse IgG (HRP-conjugated, Cell Signaling Technology, #7076, 1:10000), anti-goat IgG (HRP-conjugated, Invitrogen/Thermo Fisher Scientific, #81-1620, 1:10000) for immunoblot.

Validation

All primary antibodies in this study were purchased from commercial companies. All of these antibodies were stated to be able to detect each endogenous protein in supplier's datasheets and these antibodies were used according to supplier's protocol. AGIA antibody has been validated in published paper (Yano, et al., PLoS ONE 11: e0156716), cited in Methods. The anti-biotin goat pAb (HRP-conjugated, Cell Signaling Technology, # 7075, 1:3000) has been validated for detection of biotinylated proteins by immunoblot analysis on supplier’s website.
